# Annotations

**Published:** 1900-08-25

**Authors:** 


### Annotations.

### Plague in Europe.

The occurrence of cases of plague at the London docks and at Hamburg serves to direct renewed attention to this disease, and to give to it a personal interest which has hitherto been lacking. When one considers the constant communication between many of the greatest of our British ports and those parts of India in which for several years past plague has been more or less constantly prevalent, our freedom from it would be matter for great surprise did one not feel assured that, as an infectious disease, plague is to be placed in a very different category from many of the other infections with whose ways we are more familiar. That plague is infectious no one can doubt. In its inoculability, in the biological characters of the virus by which it can be produced, and in the fact that in fitting surroundings the disease spreads after the manner of other zymotic diseases, we have proof enough of its infectious nature. But, on the other hand, it is clear that something beyond infection is necessary for the production of anything like an epidemic. Certain it is, as is stated by Manson, that "in sanitary conditions plague does not spread even if introduced, and that in opposite conditions it may for a time spread like wildfire," but what is the particular item which makes the difference we do not know. We would not wish to dogmatise, but we would at least point out that the whole drift of modern investigations is in the direction of fixing upon our parasites much of the blame for the transference of our infections, and if it once be admitted—and there seems much ground for the admission—that when plague takes root in a community it is not a disease of man alone, but of his associated rats, and mice, and bugs, and fleas, and perhaps of other symbiotic forms of animal life, we see how idle must be those measures, on which the efforts of the sanitarians have so often been concentrated, which have had for their object the isolation of the individual human, while his multiple parasites are left to wander and to distribute the infection at their own sweet will.

It is to social and sanitary reform rather than to segregation that we must trust in fighting plague. When 300 years ago this disease receded from Western Europe, it may be presumed that it did so as the result of an improved condition of the people rather than from any systematic isolation of the sick, of which, indeed, there was none except such as consisted in daubing a yellow cross upon the door of the infected house and leaving its inmates to die or get well as might seem pleasing to the fates; and no doubt this improvement in the condition of the people has continued. On the average, there has been much progress. But in such averages we have the two extremes, and it is to be feared that while progress has made for wealth to some, and for much comfort to the majority, it has made for a depth of wretchedness to others which would hardly be beaten in the middle ages. There was a basis of truth in the juxtaposition of the words "Progress and Poverty." In the midst of our civilisation there is a residuum; and although a comparison of the conditions in which plague has been able to obtain a footing, and those in the midst of which the bulk of our population live, makes us feel very confident that plague never need be feared as seriously threatening the people of England, taking them in the mass; we cannot, on the other hand, shut our eyes to the fact that in many of our large towns there are spots in which the very conditions exist which are described by men who have worked among plague as being those most conducive to its prevalence. Dirt and overcrowding, rats and mice, abundance of body vermin and of biting parasites surely are not unknown, and that not merely in the East end, so called, but within a stone's throw of some of our best streets. Most of us will certainly go scot-free under any circumstances, but if plague should plant itself in England it will probably find plenty of victims, and it ought to be recognised beforehand that, right as it is to do everything that is possible by inspection of ships and all other such means to pick out the early cases and so prevent the disease taking root on our shores, it is not by such measures alone that it would have to be combated if once it were to obtain a footing. The unknown "something" which alone renders plague effective, and allows it to become diffused, is connected with mode of life, and will have to be fought by social reform rather than by the ambulance waggon and the isolation hospital. The lowest standard of decency and comfort which is permitted should be well above the high water mark of conditions favourable to plague.

### Crumbs from the Doctor's Table.

As the medical profession rises in its own esteem, and as its votaries aspire to be men of science, men of position, sanitarians, sociologists, and even legislators, anything, in fact, rather than mere tinkers of man's cranky frame, it is hardly to be wondered at that various classes outside the profession should be found ready to snap up any little unconsidered trifle in the way of work which the doctors may find beneath their dignity. In fact, the medical profession is the centre of a crowd of dentists, spectacle fitters, nurses, midwives, masseurs, chemists, bone setters, and bath men, all snapping, and all anxious to pick up the bits of medical and surgical work which the doctors will not take the trouble to do. And when they once get hold they do not readily let go. They soon pretend that these oddments are really their very own. Just at present the chemists are the boldest of the crew. A writer

**ANNOTATIONS**—*continued.*

in the *Pharmaceutical Journal* last week raised again the perennial cry about medical men dispensing. "Whether medical men like it or not," he says, "as they have shown themselves utterly unfit for the duties of dispensing medicine for sick and dying persons, this work must be taken from them, and handed over to those having the necessary time and skill." Then he goes on to say that "these very men," the doctors to wit, "are allowed to sign death certificates, and in that way to hide possible blunderings of their incompetent and unqualified dispensers, who may be their ignorant wives, servants, or errand boys." An old and scandalous suggestion, which we had hoped we had seen the end of. Unfortunately for the pretensions of the chemists, we find just over the page the admission that a registered chemist can act as "cover" to any number of unqualified assistants, so that the little fiction of the superior skill offered by the dispensing chemists is quickly knocked on the head. The fact is that, just as the doctors have educated themselves to such a pitch in scientific matter that many of them look down upon their ordinary work, the chemists have suffered in the same way—speaking of "the profession" of pharmacy, and pretending to look down upon the shop by which they live. One writer says that "'shop' is the great stigma that hinders advancement on the lines of scientific and artistic culture. The keeping of open shop is necessary to the public service, and must be *endured* (*sic*) at present on that account. Meantime much may be done to mitigate the *disgrace* (*sic*), &c., &c." Dear, dear! How sad it is that a chemist should have to keep a shop! And, then, these good men who loathe the "disgrace" of keeping a shop want some sort of pseudo-medical qualification, asking to be allowed "to attend cases in hospitals for specified periods, and then be given certificates, showing the holders thereof are entitled to attend the accident and poisoning cases that are brought to them, and are able to recover a proper fee for the time and attention given." They also want in place of using the inelegant term "teeth extracted" to call themselves "dental chemists." Meanwhile their trade goes to the grocers, at which they are very cross. Truly, in the upward struggle landmarks tend to be effaced.

### Our Specialists.

It has often been stated that our specialists are narrow, that they work in a groove, and that specialism tends to cramp a man's mind and give him a one-sided way of looking at things. Now, however, the specialists have cultivated alliances; no longer is an eye man content with eyes or an ear man with ears. They claim running powers over each other's lines; they deal from their "special" point of view with every organ, and by the very extension of their specialities they almost become general practitioners again. Speaking at Ipswich, Dr. Scanes Spicer, in opening the section of laryngology and otology, compared the past with the present, and said that the rapidity with which the evolution of these specialities had taken place was startling, although not to be wondered at. So long as they had limited themselves to rule-of-thumb treatment of the throat and ear, it was not surprising that they remained relatively insignificant. But now that they had found how extensive was the field embraced by diseases of the upper air passages recognition of and interest in the speciality was far more widespread. He pointed out that they had to deal with such problems as the proper growth and evolution of the skull, nose, and accessory sinuses, jaws, palate, teeth, chest, and spine, the bearing of nasal respiration on catarrhal ear processes and deafness, and the development of the orbit, the physiognomy and expression, and the normal evolution of the mental functions, as well as with the treatment of the local disease, whatever it might be. This is a large order, but it shows us the true greatness of specialism, which, indeed, is at its best when it breaks bounds, and when the knowledge gained in the persevering investigation of one region spreads through related areas and permeates the frame. Dr. Scanes Spicer's address served well to show how great is the debt of general medicine to specialism, and how untrue is the old taunt of narrowness which used to be thrown at it. Doubtless there are some who are content to use their knowledge and apply their art and go no further; to scoop out adenoids and plough turbinators, and take fees. Mere artisans and tradesmen. But the true specialist is a pioneer, always leaving behind him for the use of others a trail of knowledge gained; and nothing could illustrate the far-reaching influence of little things, and the widespread interests of what used to be regarded as but a trumpery speciality, better than the statement made by Dr. Scanes Spicer as to the extensive field of diseases coming before those who primarily have to do with nasal obstruction.

### The Health of the Navy.

Attention has been strongly drawn during the past week to the increased sick-rate from diseases of the respiratory organs which has prevailed in the Royal Navy during recent years. It would require a far more complete array of statistics than is as yet available to ascertain the cause of this unfortunate condition of affairs. Whether it is the steel vessels, as some say, or the insanitary condition of the training-ships in which, as others assert, the seeds of disease are sown, or whether it is due to the fact that nowadays our bluejackets spend more of their time ashore than they used to do, cannot be at once decided, but it certainly is a serious indictment of the service to state, as is done by Captain Ernest Rason, that in spite of better care, and being drier and less exposed than formerly was the case, the diseases of the respiratory organs in the navy have tremendously increased, and that invaliding for lung troubles has more than doubled since masts and yards have gone. Sailors on our great armour-clad vessels are no longer liable, as in the times of sailing ships, to be called from their hammocks in the middle of the night to fight for hours on the yards with wet or frozen canvas, they are not so constantly engaged keeping watch at night, they are in every way more protected, and yet so far as chest troubles go they are more diseased. Possibly it is a matter of ventilation, but in any case the increase which has taken place in respiratory troubles, while invaliding from other diseases has considerably diminished, is a matter which ought to receive the most careful attention.

